# Synapse loss in the prefrontal cortex is associated with cognitive decline in amyotrophic lateral sclerosis

**DOI:** 10.1007/s00401-017-1797-4

**Published:** 2017-12-22

**Authors:** Christopher M. Henstridge, Dimitrios I. Sideris, Emily Carroll, Sanziana Rotariu, Sally Salomonsson, Makis Tzioras, Chris-Anne McKenzie, Colin Smith, Christine A. F. von Arnim, Albert C. Ludolph, Dorothée Lulé, Danielle Leighton, Jon Warner, Elaine Cleary, Judith Newton, Robert Swingler, Siddharthan Chandran, Thomas H. Gillingwater, Sharon Abrahams, Tara L. Spires-Jones

**Affiliations:** 1 Centre for Discovery Brain Sciences, The University of Edinburgh, Edinburgh, Scotland UK; 2 Centre for Clinical Brain Sciences, The University of Edinburgh, Edinburgh, Scotland UK; 3 Department of Neurology, Ulm University, Ulm, Germany; 4 MRC Edinburgh Brain Bank, The University of Edinburgh, Edinburgh, UK; 5 Human Cognitive Neuroscience, PPLS, The University of Edinburgh, Edinburgh, UK; 6 The Euan MacDonald Centre, Edinburgh, UK; 7 UK Dementia Research Institute, The University of Edinburgh, Edinburgh, UK

## Abstract

In addition to motor neurone degeneration, up to 50% of amyotrophic lateral sclerosis (ALS) patients present with cognitive decline. Understanding the neurobiological changes underlying these cognitive deficits is critical, as cognitively impaired patients exhibit a shorter survival time from symptom onset. Given the pathogenic role of synapse loss in other neurodegenerative diseases in which cognitive decline is apparent, such as Alzheimer’s disease, we aimed to assess synaptic integrity in the ALS brain. Here, we have applied a unique combination of high-resolution imaging of post-mortem tissue with neuropathology, genetic screening and cognitive profiling of ALS cases. Analyses of more than 1 million synapses using two complimentary high-resolution techniques (electron microscopy and array tomography) revealed a loss of synapses from the prefrontal cortex of ALS patients. Importantly, synapse loss was significantly greater in cognitively impaired cases and was not due to cortical atrophy, nor associated with dementia-associated neuropathology. Interestingly, we found a trend between pTDP-43 pathology and synapse loss in the frontal cortex and discovered pTDP-43 puncta at a subset of synapses in the ALS brains. From these data, we postulate that synapse loss in the prefrontal cortex represents an underlying neurobiological substrate of cognitive decline in ALS.

**Electronic supplementary material:**

The online version of this article (10.1007/s00401-017-1797-4) contains supplementary material, which is available to authorized users.

## Introduction

Amyotrophic lateral sclerosis (ALS) is the most common form of motor neurone disease (MND), resulting in progressive upper and lower motor neurone dysfunction and loss. This leads to progressively severe muscle wasting and most patients succumb due to respiratory failure, with a mean survival time of 3 years post diagnosis [57]. Due to the striking, and in many cases rapid, deterioration of motor function, ALS was historically considered a homogenous disease of the motor system. However, both clinical and post-mortem studies increasingly describe ALS as a heterogeneous disorder, with around 50% of patients exhibiting neuropsychological deficits due presumably to breakdown of non-motor areas of the brain [49]. ALS is thought to lie on a disease spectrum with frontotemporal dementia (FTD). These seemingly disparate disorders share both genetic and pathological features [30] with considerable clinical overlap. An estimated 15% of FTD patients present with motor decline meeting the criteria for ALS diagnosis and 15% of ALS cases show severe cognitive and or behavioral decline, meeting the FTD diagnostic criteria [24, 30, 49]. However, a further 30–40% of ALS patients show milder, more specific cognitive and/or behavior changes (ALS cognitive impairment—ALSci/ALS behavioral impairment ALSbi [49]) that does not meet FTD diagnostic criteria but can still affect daily life [20]. Changes include executive dysfunction, decreased verbal fluency, language impairment and social cognition deficits, which are thought to be mediated by regions of the frontal cortex and are similar to mild FTD-associated impairments [20]. Behavior change is predominantly one of the apathies, while other features of FTD are also seen, including disinhibition and loss of sympathy/empathy [20]. Functional and structural imaging studies support the presence of frontal deficits [11, 34, 47, 56], and specifically relate dysfunction of the dorsolateral prefrontal cortex [including Brodmann Area 9 (BA9)] to the cognitive profile in ALS [1, 2]. It is vitally important that we understand the underlying neuropathology driving these cognitive deficits, as cognitively impaired patients not only endure the personal burden of both motor and cognitive dysfunction, but also exhibit a shorter survival time from symptom onset [15, 58].

Synapse degeneration is a common feature in a large number of neurodegenerative diseases and often plays a role in pathogenesis, rather than simply occurring as a secondary effect of neuron loss [22]. For example, synapse loss remains the strongest neurobiological correlate of cognitive decline in Alzheimer’s disease (AD [54]). In AD, synaptic accumulation of key pathological forms of tau and beta-amyloid are thought to drive synaptic breakdown [48]. Likewise, studies in ALS mouse models have suggested that synapse defects in motor regions of the cortex are an early/pre-symptomatic feature of the disease [18, 43]. However, to date, few studies have attempted to assess the contribution of synaptic change to pathology of non-motor cortical regions in the brain of human ALS patients and none using direct measurement in patients with defined ALS with cognitive impairment.

By combining the high-resolution imaging techniques of array tomography and transmission electron microscopy (TEM), we have examined synapses in the frontal cortex and motor cortex of cognitively unimpaired and cognitively impaired ALS (ALSci) patients, to define the relationship between synaptic breakdown and cognitive impairment. Cognitive profiling was performed using the Edinburgh Cognitive and Behavioural ALS Screen (ECAS) [3], a short multi-domain assessment designed for people with physical disability which has been validated against extensive neuropsychology [38]. Other post-mortem measurements such as cortical thickness, pathological burden and gliosis were recorded to ensure a comprehensive profiling of each case.

## Materials and methods

### Participant characteristics

All patients had clinical and electrophysiological evidence of combined upper and lower motor neurone involvement and fulfilled the revised El Escorial criteria for a diagnosis of ALS [10]. Patients were recruited through the Scottish Motor Neurone Disease Register and data collected in the CARE-MND database (Clinical Audit Research and Evaluation). Ethical approval for this register was obtained from Scotland A Research Ethics Committee 10/MRE00/78 and 15/SS/0216. All clinical data were subsequently extracted from the CARE-MND database. Clinical details can be seen in Table 1. There was no documented evidence of dementia in the CARE-MND database.

**Table 1:** Summary demographics of all donors

	Number	Male/female	Age at diagnosis (median + range)	Age at PM (median + range)	Site of onset	Riluzole (yes/no)	Disease duration (months)	Time between ECAS and death (months)	ECAS total (mean ± SD)	ALS-specific ECAS (mean ± SD)
**Cohort 1-EM**										
Control	5	4/1		61 (53–77)						
ALS	20	9/11		63 (40–89)						
**Cohort 2a-array tomography**										
Control	14	11/3		79 (62–95)						
ALS	29	18/11	60 (32–75)	64 (40–89)	20 Limb, 7 bulbar	17/9	44 (17–216)			
**Cohort 2b (subset of cohort 2)-cognitive profiling**										
Control	14	11/3		79 (62–95)						
ALS	16	9/7	60 (32–71)	64 (40–83)	11 Limb, 5 bulbar	4/6	39 (24–216)	17 (4–42)	116 ± 4.9	86 ± 3.6
ALSci	7	4/3	61 (38–72)	68 (43–75)	4 Limb, 2 bulbar	4/3	49 (34–64)	14 (3–61)	98 ± 5.9	72 ± 7.1
**Cohort 3-CSF**										
ALS	9	6/3		58 (41–69)	3 Limb, 1 bulbar, 5 mixed (bulbar and limb)		28 (12–84)		112.6 ± 5.5	
ALSci	6	4/2		72 (65–80)	5 Limb, 1 bulbar		15 (1–36)		62.3 ± 16.6	

*PM* post-mortem, *ECAS* Edinburgh Cognitive and Behavioral ALS Screen, *EM* electron microscopy, *ALSci* ALS with cognitive impairment

Patients gave pre-mortem consent for brain and spinal cord donation. All tissue retrievals were in line with the Human Tissue (Scotland) Act, and all procedures have been approved by a national ethics committee. Use of human tissue for post-mortem studies has been reviewed and approved by the Sudden Death Brain Bank ethics committee and the ACCORD medical research ethics committee, AMREC (ACCORD is the Academic and Clinical Central Office for Research and Development, a joint office of the University of Edinburgh and NHS Lothian). Further, some brains from human subjects with no cognitive deficits were obtained through the Massachusetts Alzheimer’s Disease Research Centre and Massachusetts General Hospital Neuropathology department. All donor tissue was obtained in accord with local and National Institutional Review Board regulations. Details of all donors used in this study can be found in Supplementary Table 1, with means for each group found below in Table 1.

At post-mortem, the brain was removed as detailed in [46] and cut into coronal slices. Regions of interest were then dissected from each coronal slice. Each region of interest from one hemisphere was then processed for paraffin embedding. Samples from the other hemisphere were dissected into smaller segments then processed for array tomography or transmission electron microscopy (see below for details).

### Cognitive profiling

The ECAS is a brief multi-domain assessment which is specifically designed for ALS. It includes the assessment of domains which are typically affected in ALS (Fluency, Executive and Language functions) and which together produce an ALS-Specific Score (max 100). It also includes the assessment of domains which are more typically affected in other disorders such as Alzheimer’s disease (Memory, Visuospatial Functions) and which make up the ALS non-specific score (max 36). Together, the combined ALS-specific and ALS non-specific scores produce an ECAS total Score (max 136). The assessment has been validated against extensive neuropsychology and provides high sensitivity and specificity using published cut-offs with patients classified as ALSci if ALS-specific and/or ECAS total Scores are below threshold [38]. It also shows good convergent validity with other screens not designed for ALS [31, 41].

### Genetics

A subset of our patient participants actively consented to donate blood and saliva to the Scottish Regenerative Neurology Tissue Bank for genetic analysis. Use of patient samples for genetic profiling has been approved by the Chief Scientist Office Scotland; MREC/98/0/56 1989–2010, 10/MRE00/77 2011–2013, 13/ES/0126 2013–2015, 15/ES/0094 2015-present. *C9orf72* genotyping was performed as described in [12] and most cases were genetically analyzed as described in [5]. Some cases were analyzed by next generation sequencing to screen for variants in the coding regions of *SOD1*, *TARDBP*, *FUS*, *VCP*, *UBQLN2*, *SQSTM1*, *CHMP2B* and *VAPB*. Library construction was carried out using the Ion Ampliseq library kit v2.0 (Life Technologies). Emulsion PCR and enrichment was performed using the Ion OneTouch2 instrument (200p template kit) and Ion ES module. Sequencing was carried out on an Ion Torrent Personal Genome Machine (Life Technologies) using the Ion PGM sequencing 200 kit v2 and an Ion 316 chip. All stages followed the manufacturer’s protocols. NextGENe software (Softgenetics) was used with hg19 (GRCh37) human genome as reference to identify variants.

### Neuropathology

Fresh post-mortem tissue blocks were fixed in 10% formalin for a minimum of 24 h. Tissue was dehydrated in an ascending alcohol series (70–100%), followed by three xylene washes, all for 4 h each. Next, three paraffin waxing stages (5 h each) were performed to ensure full penetration of the embedding wax and the blocks were cooled. Tissue sections were cut on a Leica microtome at 4 μm and collected on glass slides. All sections were dried at 40 °C for at least 24 h before staining. Immunohistochemistry was performed using standard protocols, enhanced using the Novolink Polymer detection system and visualized using DAB as chromogen. See Table 2 for antibody information. Slides were finally counterstained with hematoxylin for 30 s to stain cell nuclei. For each staining run, positive and negative control tissue for each pathological marker were used to ensure specificity and success of each experiment. All staining was performed by experimenters blind to clinical diagnosis.

**Table 2:** Primary antibody information

Histopathology				
Antibody	Company	Code	Dilution	Pre-treatment
Beta amyloid (BA4)	Dako	M087201-2	1:100	98% Formic acid 5 min
pTDP-43	2B Scientific	CAC-TIP-PTD-MO1	1:4000	Pressure cooker/citric acid
pTau (AT8)	Thermo	MN1020	1:2500	None
GFAP	Dako	Z0334	1:800	None
CD68	Dako	M0876	1:100	Pressure cooker/citric acid

Array tomography				
Antibody	Company	Code	Dilution	Secondary antibody
Synaptophysin	Abcam	Ab8049	1:50	Donkey α Mouse—AF594 or AF488
PSD95	Synaptic Systems	124,014	1:50	Donkey α Guinea Pig—AF647
pTDP-43	Proteintech	22309-1-AP	1:50	Donkey α Rabbit—AF594 or AF488

For pTDP-43 and pTau analysis, cases were described as being positive or negative depending on the presence of clearly stained protein aggregates.

### Burden quantification

All sections stained for β-amyloid, CD68 or GFAP was assessed for plaque, microglia and astrocytic burden, respectively, using Stereo Investigator. Cortical gray matter was outlined in each section and immune-positive objects identified using an automated color-based thresholding algorithm. The area of immuno-positive cortex was expressed as a percentage of total cortex in each brain region. All imaging and analysis were performed by experimenters blind to clinical diagnosis.

### Cortical thickness

Gray matter thickness was calculated at ten randomly selected points throughout each section. Thickness was measured from the pial surface to the border of grey/white matter, below cortical layer 6. All ten measurements were averaged to give a cortical thickness for each brain region. All imaging and analysis weres performed by experimenters blind to clinical diagnosis.

### Neuron counts

Paraffin-embedded sections from the frontal cortex of 10 ALS cases were used to assess neuronal density as previously described [21]. The five ALS cases with the highest and the five with the lowest synapse density as measured by array tomography were analyzed. All imaging and analysis were performed by experimenters blind to clinical diagnosis.

### Neurofilament light-chain CSF ELISA

Fifteen patients provided CSF for this study. Patients were seen at the Department of Neurology in Ulm. The study, whose overall aim was to find biomarkers for neurological diseases, was approved by the local ethics committee in Ulm according to institutional guidelines and patients provided written informed consent to participate (approval number 20/10). A summary of patient group characteristics is given in Supplementary Table 1. The physical function of ALS patients’ status was classified with the revised ALS functional rating scale (ALSFRS-R) with a maximum of 48 points, where lower values represent a more severe disease stage.

CSF was obtained by lumbar puncture, centrifuged, aliquoted and stored within 2 h at − 80 °C until analysis. Neurofilament light-chain levels in the CSF were examined using a commercial 96-well ELISA kit from UmanDiagnostics (Umea, Sweden). CSF samples were diluted 1:20 in sample diluent before analysis and the ELISA was run according to the manufacturer’s instructions, with all CSF samples and kit standards run in duplicate.

### Electron microscopy

Fresh post-mortem samples, stored in 0.1 M PB were trimmed into small cortical blocks and fixed in 4% paraformaldehyde and 2.5% glutaraldehyde in 0.1 M PB for 3 h. Fixed blocks were washed twice in 0.1 M PB before being cut at 70 μm with a vibratome. Sections were then treated with osmium tetroxide (1% in 0.1 M PB) for 30 min (protected from light) and dehydrated in an ascending series of ethanol and propylene oxide, before embedding in Durcupan resin. During dehydration, the sections were treated with uranyl acetate (1% in 70% ethanol) for 40 min in the dark. Durcupan resin was polymerized for 48 h at 56 °C. Small regions of interest were cut from the Durcupan-embedded sections and glued onto Durcupan blocks, before cutting into 70 nm ribbons using an ultracut microtome (Leica) with a Jumbo Histo Diamond Knife (Diatome, Hatfield, PA). Ribbons were collected on grids and stained with lead citrate before imaging on a Philips CM12 transmission electron microscope equipped with a Gatan digital camera. Qualitative and quantitative synapse analyses were performed by an investigator who was blinded to the disease status of each sample. Individual synaptic profiles were identified based on the presence of clear pre- and post-synaptic terminals, containing synaptic vesicles and post-synaptic density, respectively. Degenerating synapses were classified as those with an electron-dense cytoplasm, as previously described [19, 26].

### Array tomography

Brain samples were prepared for array tomography as outlined previously [26]. Briefly, fresh post-mortem tissue was trimmed into small cortical blocks and fixed in 4% paraformaldehyde and 2.5% sucrose in PBS for 2–3 h. Samples were then dehydrated through ascending ethanol washes and into LR White resin (Electron Microscopy Sciences) overnight. Blocks were placed individually into capsules containing LR White and polymerized overnight at 53 °C. Tissue blocks were cut into ribbons of 70 nm serial sections using an ultracut microtome (Leica) with a Histo Jumbo Diamond Knife (Diatome, Hatfield, PA) and collected on gelatin-coated glass coverslips. For synaptic density analysis, ribbons were immunostained as described previously [21], with a primary antibody against synaptophysin overnight (see Table 2 for antibody details) and no primary negative control was included to rule out non-specific binding of the secondary antibodies. The following day, the staining was developed with a fluorescently labeled secondary antibody (1:50 dilution) and finally mounted onto slides with Immu-mount mounting media. To obtain improved staining with the pTDP-43 antibody, array ribbons underwent epitope retrieval. Ribbons were pressure-cooked for 2 min in citrate buffer (pH 6), and then allowed to cool to room temperature. Ribbons were washed with TBS (2 × 5 mins) and then underwent the same staining protocol as described above. Three antibodies were used in combination (see Table 2 for details), to analyze the presynapse (synaptophysin), postsynapse (PSD95) and pTDP43 co-localization. Once the ribbons had been stained and mounted, images were obtained at the same position on each section along the ribbon, using a Zeiss axio Imager Z2 epifluorescent microscope equipped CoolSnap digital camera and AxioImager software with array tomography macros (Carl Zeiss, Ltd, Cambridge UK). First, a tile scan of the entire ribbon was obtained at 10× magnification; then regions of interest were chosen on the tilescan and a position list generated. High-resolution images were obtained from the position list with a 63× 1.4 NA Plan Apochromat objective. At least two image stacks were captured from at least two tissue blocks per region, per case. This meant at least four image stacks were captured per region for each case.

Stacks were aligned using the MultiStackReg ImageJ plugin [55]. Regions of interest (ROI) were automatically chosen in the neuropil of each aligned image stack using an in-house MATLAB cropping script and coded for blind analysis, before thresholding with an automated algorithm in ImageJ. To remove false background staining which was only found on single sections, we used the Watershed script [35] which also provides 3-dimensional (3D) object counts. These 3D objects (synaptic puncta) were used to generate synapse densities (synaptic puncta per mm^3^) based on the ROI volume. All individual ROI synapse densities (between 100 and 300 ROIs per region, per case) from each region, from each case were combined and a mean density value generated. All imaging and analysis were performed by experimenters blind to clinical diagnosis.

### Statistics

GraphPad Prism 7.02 was used to test data for normality (D’Agostino and Pearson normality test). A mean or median for each subject was calculated for each measurement, following which parametric (*t* test or ANOVA) or non-parametric (Mann–Whitney or Kruskal–Wallis) tests were performed as required. Significance was reported when *p* < 0.05. Data are usually presented as mean ± standard deviation, unless otherwise stated.

### Data sharing

Data used in this manuscript including custom image analysis scripts for ImageJ will be freely shared on the University of Edinburgh Data Repository. Analysis macros can be found at the following link: https://dx.doi.org/10.7488/ds/2268. Original raw image files can be found at the following link: 10.7488/ds/2269.

## Results

**Fig. 1:**
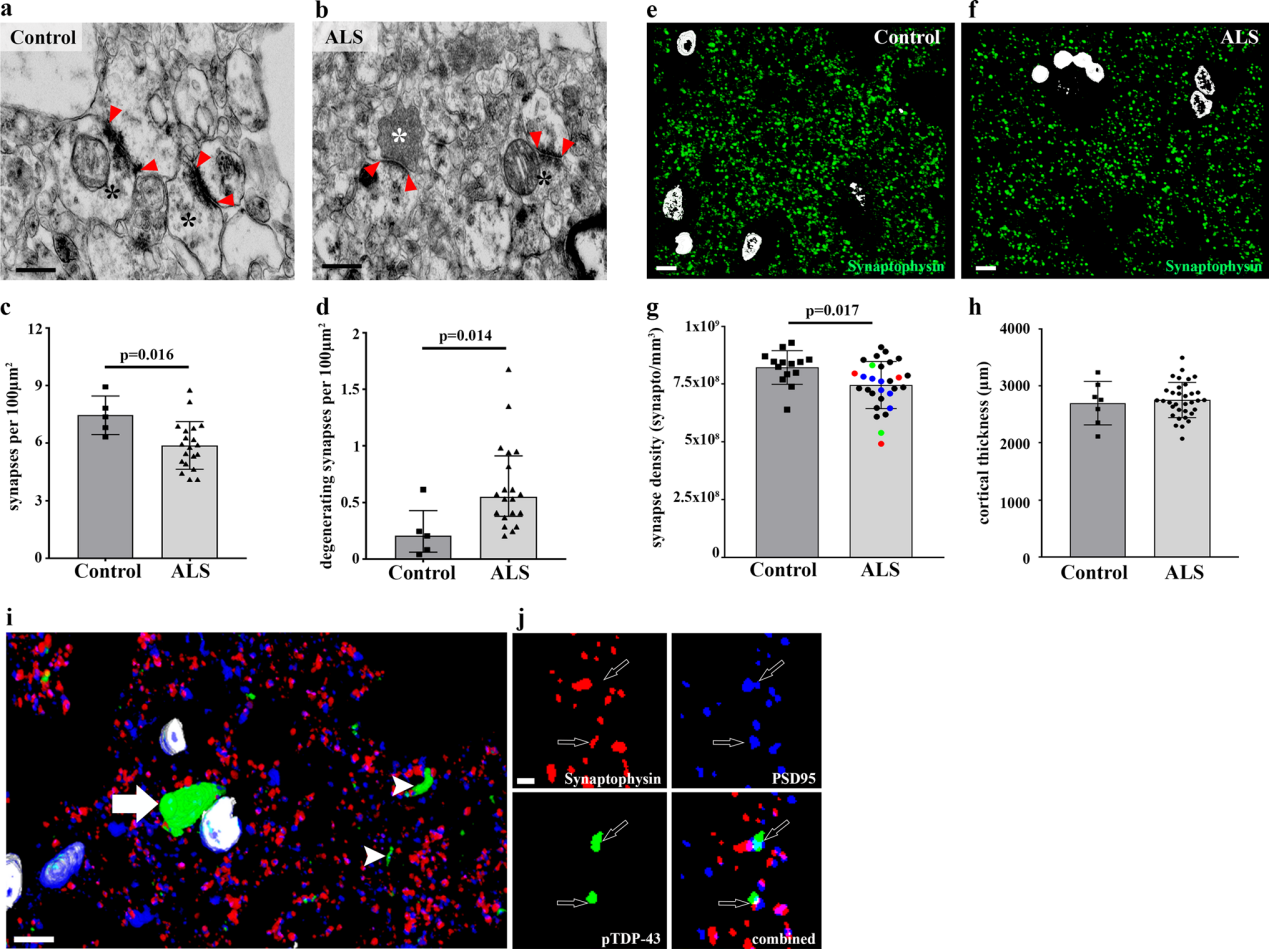
Synapse loss in ALS frontal cortex. Representative electron micrographs showing two excitatory synapses in the frontal cortex of a healthy control (**a**) and an ALS case (**b**). Edges of the post-synaptic density (PSD) are marked by red arrowheads and pre-synaptic terminals by black asterisks. The white asterisk in **b** highlights an electron dense, degenerating presynaptic terminal. Scale bars 100 nm. Decrease in synaptic density in the frontal cortex [(**c**) 2-tailed unpaired *t* test; *p* = 0.016], and increased numbers of degenerating synapses [(**d**) 2-tailed Mann–Whitney test; *p* = 0.014] in ALS patients (*n* = 20) compared to controls (*n* = 5), measured using TEM. Each data point represents the mean total synapse (**c**) or degenerating synapse (**d**) count per 100 µm^2^ for each individual. Three-dimensional reconstructions of fifteen 70 nm array tomography sections from a control frontal cortex (**e**) and an ALS frontal cortex (**f**), stained for synaptophysin (green) and DAPI (white). Scale bar is 10 µm. **g** Decrease in synaptic puncta (2-tailed unpaired *t* test; *p* = 0.017) in ALS patients compared to controls, measured using array tomography. Each data point represents the mean synapse count per mm^3^ of frontal cortex, for each individual (control *n* = 14; ALS *n* = 29). Blue icons = *C9orf72* expansions, Red icons = *SOD1* variant and Green icons = *NEK1* variant. **h** Cortical thickness comparisons between control (*n* = 7) and ALS (*n* = 34) revealed no difference (2-tailed unpaired *t* test; *p* = 0.76). Each data point represents the mean of ten measurements per cortical block. All histogram bars plot the mean ± SD. **i** 3D reconstruction of 25 array tomography sections (each 70 nm thick), stained for synaptophysin (red), PSD95 (blue), pTDP-43 (green) and DAPI (white). A large perinuclear pTDP-43 aggregate (white arrow), numerous pTDP-43 + ve neuritic threads (white arrowheads) and small pTDP-43 puncta (open arrows) are clearly visible. Scale bar 10 µm. By cropping the image in **i**, compressing 3 consecutive sections and then splitting the channels, it is evident in panel (**j)** that pTDP-43 aggregates are found both in some pre (synaptophysin, red) and post (PSD95, blue) synaptic compartments. Scale bar 1 µm

Transmission electron microscopy (TEM) analysis of the prefrontal cortex [Brodmann Area 9 (BA9); ~ 150 synapses per case] from 5 control and 20 ALS cases (Table 1; cohort 1) revealed a significant decrease in synaptic density in the ALS cohort (Fig. 1a–c). In addition, there was a significant increase in the number of electron-dense, degenerating pre-synaptic terminals in ALS cases compared to controls (Fig. 1b, d) [19].

To expand our synaptic analyses, we next examined a largely independent cohort (Table 1; cohort 2a) of subjects using higher-throughput array tomography. Analysis of over 1 million synapses (~ 36,000 synapses per case) from 14 controls and 29 ALS brains using array tomography confirmed our initial TEM findings revealing synapse loss in BA9 of ALS patients (2-tailed unpaired *t* test; *p* = 0.013; Fig. 1e–g). Post-mortem interval (PMI) did not influence synapse density (Pearson *r* = − 0.21, *p* = 0.17). Importantly, the loss of synaptic density was not accompanied by any detectable gross atrophy of BA9 (2-tailed unpaired *t* test; *p* = 0.68; Fig. 1h). Furthermore, stereological estimates of neuron density counts from 5 cases with the highest and lowest synapse densities were not significantly different (Low: *n* = 5, 23,370 ± 2756 neurons/mm^3^; High: *n* = 5, 28068 ± 2094 neurons/mm^3^. Unpaired 2-tailed *t* test; *p* = 0.22), suggesting that synapse loss was occurring in the absence of concomitant, overt neuronal loss.

We then performed some exploratory analyses to assess the influence of numerous genetic and pathological factors that may associate with increased synapse loss. Genetic screening revealed that 6 ALS cases had a hexanucleotide repeat expansion in *C9orf72*, 3 cases had *SOD1* variations and 2 had *NEK1* variations (Supplementary Table 1). However, we did not observe any significant association between lower synapse density in BA9 and distinct genetic mutations in ALS with this sample size (Fig. 1g; Supp. Fig. 1).

Pathological changes in disease-associated proteins are strongly implicated in driving synapse degeneration in dementias [48]. Thus, we next assessed the presence of disease-associated pathological aggregates in formalin-fixed paraffin-embedded (FFPE) sections from BA9 and their association with synapse loss in array tissue from the same case. We found no association between the presence of beta-amyloid or pTau pathology in BA9 and synapse loss (Supp. Fig. 2). We also found no association between the presence of pTDP-43 aggregates in BA9 and synapse loss (Supp. Fig. 3). However, it is known that ALS patients with *SOD1* mutations do not express pTDP-43 pathology [32]. When we remove our three SOD1 + ve cases from the analysis, we find a significantly lower synapse density in ALS cases with pTDP-43 pathology (Supp. Fig. 3). Due to this potential association and to the literature from Alzheimer’s disease in particular, indicating that pathological protein accumulation at synapses can drive synapse degeneration [48], we used array tomography to assess the presence of pTDP-43 at the synapse. We observed pTDP-43 pathology in all of the 9 ALS cases screened, often exhibiting as large cytoplasmic aggregates or neuritic threads (Fig. 1i). Interestingly, we also noticed small pTDP-43 puncta in the neuropil of our 9 cases, which in a subset of synapses co-localized with synaptic markers (Fig. 1i, j).

**Fig. 2:**
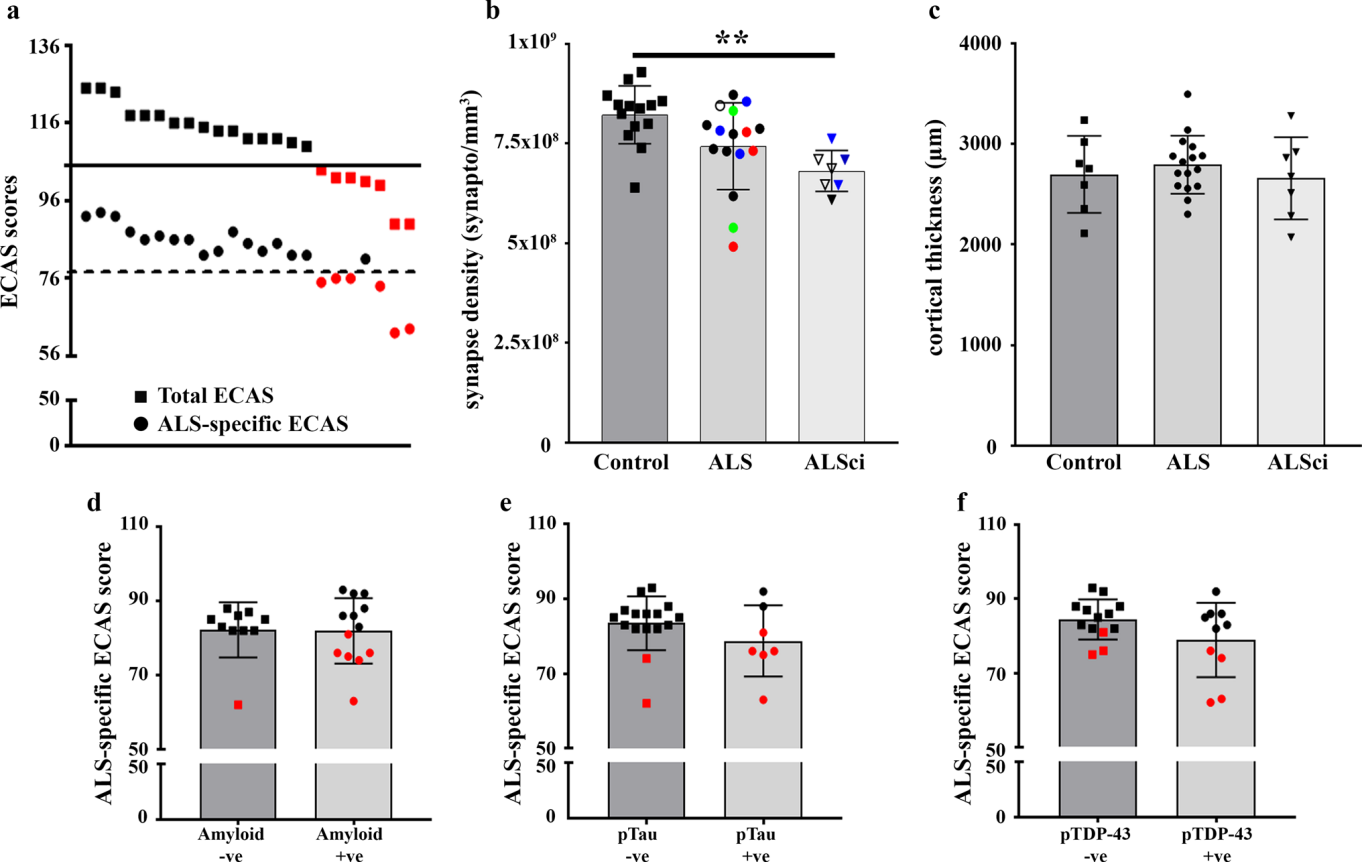
Synapse loss in the ALS frontal cortex associates with cognitive decline. **a** Graph showing all 23 cognitively profiled cases and their total ECAS scores (squares) and ALS-specific ECAS scores (circles). Solid black line represents the performance threshold for abnormality for total ECAS score (105) and the dashed line for ALS-specific ECAS score (77). This reveals 7 cases fell below the total ECAS threshold, indicating cognitive impairment and 6 of those cases fell below the ALS-specific threshold (red shapes). **b** Histogram showing synapse density in the frontal cortex of control, ALS unimpaired (ALS) and ALS impaired (ALSci) cases. One-Way ANOVA revealed a significant difference between the groups (*F* = 6.6; *p* = 0.004) and the Tukey post hoc test revealed a significant difference between control and ALSci (*p* < 0.01). Open shapes = cases not genotyped, Blue icons = *C9orf72* expansions, Red icons = *SOD1* variant and Green icons = *NEK1* variant. **c** Cortical thickness did not differ between any of the groups (One-Way ANOVA; *F* = 0.46, *p* = 0.64). The presence of amyloid (**d**), pTau (**e**) or pTDP-43 (**f**) had no effect on cognitive score (2-tailed unpaired *t* tests; *p* > 0.05). Red icons = ALSci. All histogram bars plot the mean ± SD

Twenty-three patients from whom we collected tissue for array tomography had previously been cognitively profiled using the Edinburgh Cognitive and Behavioural ALS Screen (ECAS). These individuals (Table 1; cohort 2b) were classified as unimpaired (ALS) or ALS with cognitive impairment (ALSci) according to established threshold scores for ECAS Total or ALS-specific ECAS results [3]. The scores revealed 16 unimpaired cases and 7 impaired cases [70% unimpaired, 30% impaired (Fig. 2a)]. Comparing ECAS data with synaptic data, we found a significant difference between our three groups in synaptic density and that the cognitively impaired group had a significantly lower synapse density in BA9 than controls (Fig. 2b). We found no significant difference between the ALS unimpaired cohort and either controls or ALSci, most likely due to two cases with very low densities in the unimpaired group (Fig. 2b). When we plotted ECAS score versus synapse density, we found no significant correlation between these data (Pearson *r* = 0.31, *p* = 0.15), again due to the influence of the two unimpaired cases with low synapse density mentioned above (Supp. Fig. 4). One potential confounding factor is the length of time between cognitive testing and post-mortem synapse analysis. However, we found no correlation between synapse density at post-mortem and time since cognitive testing (Pearson *r* = 0.043, *p* = 0.85). Although there was no association between genetic status and synapse density or cognitive impairment, it was interesting to note that 3 of our 4 genetically screened cognitively impaired cases were positive for *C9orf72* repeat expansions (Fig. 2b), warranting further study of the effects of *C9orf72* expansions, as more cases prepared for these techniques become available. Cortical thickness did not vary between the groups (ALS, ALSci, Controls) (Fig. 2c), suggesting that atrophy and overt neuron loss were not driving cognitive decline. We found no association between age and ECAS score (Supp. Fig. 5) or cortical thickness and ECAS score (Supp. Fig. 5). Furthermore, the presence of beta-amyloid or pTDP-43 pathology in BA9 did not associate with lower ECAS score (Fig. 2d, f). Interestingly, although the presence of pTau pathology did not associate with a lower mean ECAS score (Fig. 2e), pTau + ve cases were predominantly ALSci (5 of the 7 pTau + ve cases were ALSci), whereas pTau-ve cases were predominately unimpaired ALS cases (12 of the 14 pTau-ve cases were unimpaired ALS; Fisher’s exact test: *p* = 0.005). This suggests that pTau pathology may associate with cognitive impairment and warrants further study once more cases become available.

Eleven of the ECAS-screened cases also underwent the ECAS behavior interview, which was completed by an informant/carer in vivo. Six cases were unimpaired and five were classified as ALS with behavioral impairment (ALSbi), with changes including apathy, lack of empathy and behavioral disinhibition. Analysis revealed no difference in BA9 synapse densities between the three groups of controls, ALS and ALSbi (One-way ANOVA; *F* = 1.93; *p* = 0.17).

Together, these data suggest that synapse density is a robust indicator of ECAS score and cognitive performance in ALS.

**Fig. 3:**
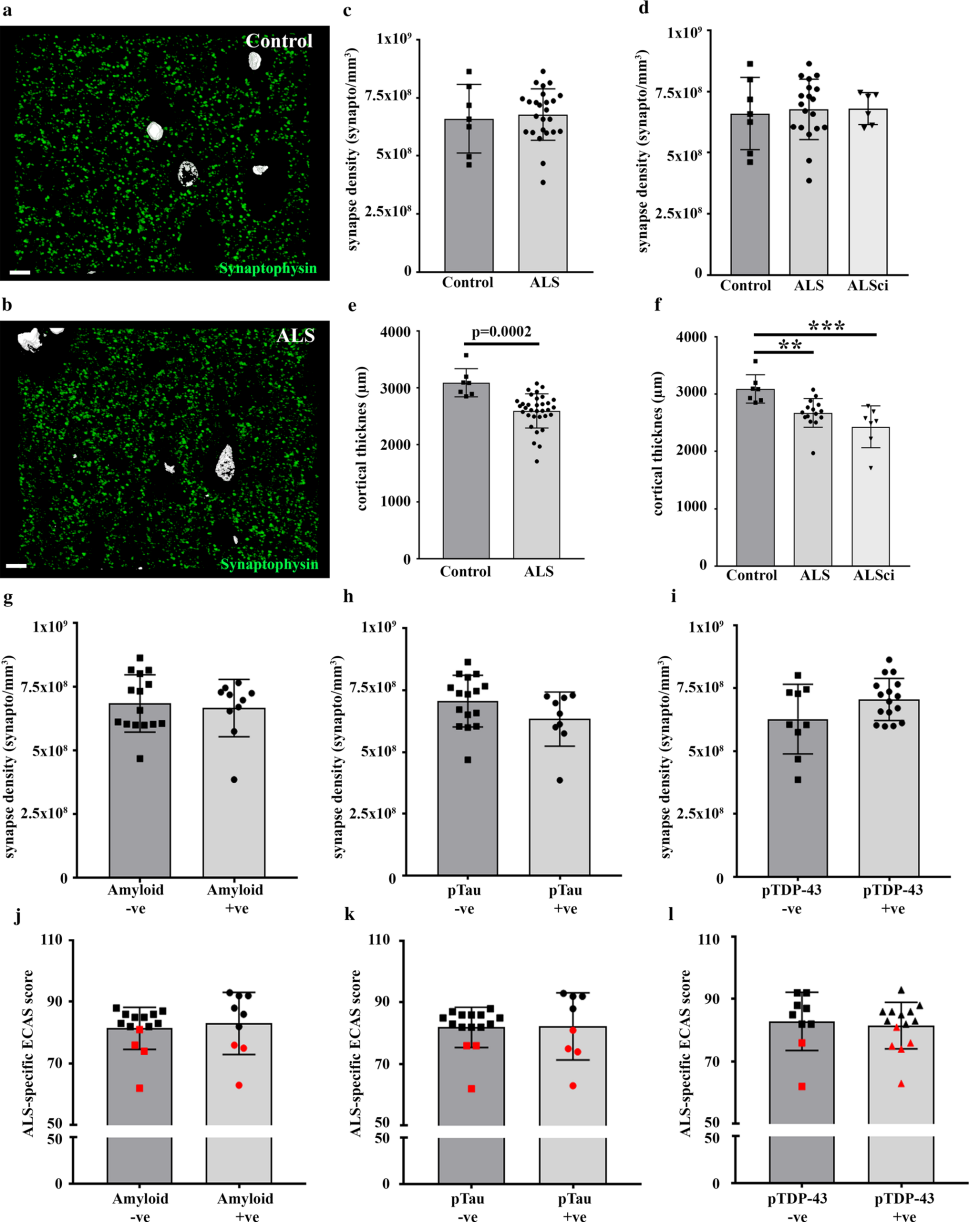
Synapse density in the ALS motor cortex does not associate with cognitive decline. Three-dimensional reconstruction of twenty-four 70 nm array tomography sections from a control motor cortex (**a**) and an ALS motor cortex (**b**), stained for synaptophysin (green) and DAPI (white). Scale bar is 10 µm. **c** Histogram showing no change in synaptic puncta within the motor cortex (2-tailed unpaired *t* test; *p* = 0.73) between control and ALS. Each data point represents the mean synapse count per mm^3^ of motor cortex, for each individual (control *n* = 7; ALS *n* = 25). **d** When split by cognitive status, there is no difference in synapse density within the motor cortex (One-Way ANOVA; *F* = 0.06, *p* = 0.94). **e** Histogram showing a significant decrease in the thickness of ALS motor cortex compared to controls (2-tailed unpaired *t* test; *p* = 0.0002). **f** Both ALS and ALSci motor cortices are significantly thinner than control (One-Way ANOVA; *F* = 9.98, *p* = 0.0006; Tukey post hoc test; *p* < 0.05 and *p* < 0.01, respectively). The presence of beta-amyloid (**g**), pTau (**h**) and pTDP-43 (**i**) in the motor cortex, had no effect on synapse density (2-tailed unpaired *t* tests; *p* > 0.05). The presence of beta-amyloid (**j**), pTau (**k**) and pTDP-43 (**l**) in the motor cortex, had no effect on cognitive score (2-tailed unpaired *t* tests; *p* > 0.05). Red icons = ALSci. All histogram bars plot the mean ± SD

To address whether synapse breakdown, in the absence of cortical atrophy, is a global feature of pathology in the ALS brain, or a specific event occurring in the prefrontal cortex, we employed an array tomography approach to analyze ~ 750,000 synapses from the primary motor cortex. Comparing synapse density in 7 controls versus 25 ALS patients, we discovered no difference in synapse density (Fig. 3a–c). Grouping the BA4 synapse values based on cognitive status did not reveal any difference in synapse density between the groups (Fig. 3d). However, there was significant cortical thinning of the motor cortex in ALS patients compared to controls (Fig. 3e). When the ALS group was divided into unimpaired ALS and ALSci, it was found that both groups exhibited significant cortical atrophy compared to controls (Fig. 3f). Furthermore, the assessment of neurofilament light-chain in the cerebrospinal fluid (CSF) of another cohort of ALS and ALSci patients (Table 1; cohort 3) revealed comparable levels (ALS = 7950 ± 2030 pg/ml, ALSci = 11,068 ± 3073 pg/ml. 2-tailed unpaired *t* test; *p* = 0.39), suggesting the presence of similar neuronal breakdown across the brain in both groups (Supp. Fig. 6). When comparing synapse density in the frontal cortex to the motor cortex within the healthy control brains, we discovered a lower density in the motor cortex (Supp. Fig. 7; 2-tailed paired *t* test *p* = 0.036). This difference in regional synapse density was also observed in the larger group of cognitively unimpaired ALS cases (Supp. Fig. 7; 2-tailed paired *t* test *p* = 0.005), but was lost in the ALSci cases (Supp. Fig. 7; 2-tailed paired *t* test *p* = 0.75). This may suggest that synapse densities in the frontal cortex of ALSci brains had dropped to levels similar to those found in the motor cortex. We found no association between beta-amyloid, pTau or pTDP-43 pathology in BA4 and synapse density (Fig. 3g–i). Further, we found no association between beta-amyloid (Fig. 3j), pTau (Fig. 3k) or pTDP-43 (Fig. 3l) pathology in the motor cortex and cognitive score.

Glial cells are thought to play a prominent role in ALS pathogenesis [40], so we analyzed GFAP and CD68 burdens as a measure of astrocyte and microglial activity, respectively, in both BA9 and BA4. We found no correlation between GFAP burden and synapse density (Supp. Fig. 8) in BA9 (Pearson *r* = 0.127, *p* = 0.51) or in BA4 (Pearson *r* = 0.16, *p* = 0.44). There was also no correlation between CD68 burden and synapse density (Supp. Fig. 8) in BA9 (Pearson *r* = − 0.012, *p* = 0.95) or BA4 (Pearson *r* = 0.036, *p* = 0.86). Furthermore, there was no difference in GFAP or CD68 burden in BA9 (Supp. Fig. 8) in ALS patients compared to ALSci (2-tailed unpaired *t* tests. GFAP: *p* = 0.09; CD68: *p* = 0.59). This suggests that synapse loss in the ALSci patients is not associated with altered gliosis.

Taken together, these data suggest that synapse degeneration, occurring specifically within frontal (and not motor) cortex of ALS patients with cognitive deficits, is one underlying neurobiological contributor to cognitive decline. Of all of the pathological changes measured, synapse degeneration in BA9 was the strongest predictor of cognitive impairment.

## Discussion

We have optimized a combination of cognitive and genetic profiling with post-mortem tissue collection, neuropathology and high-resolution subcellular analyses. Our human tissue collection and processing protocol [26] robustly preserves synaptic structures for both electron microscopy and array tomography analysis. To the best of our knowledge, this is the only ALS cohort in the world that has been extensively characterized to this level of detail.

A few previous studies attempted to assess synaptic changes in human post-mortem tissue using less direct measurements such as optical density of immunohistochemical staining or western blotting [25, 45], but failed to observe any difference between ALS patients and controls. However, one potential confound is that we do not know the cognitive status of the patients in these studies [25, 45] and taken together our study highlights the importance of using highly precise, high-resolution approaches (such as array tomography and TEM) when studying synaptic changes in post-mortem material. One very recent study has detected a loss of synaptic terminals in the putamen and caudate of ALS patients, using immunohistochemical approaches [44]. Patients with severe cognitive decline meeting the diagnosis of FTD or FTD-MND had significant synaptic loss, whereas the ALS group had a more variable phenotype. Unfortunately, the ALS group was not stratified by cognitive status, so we cannot discern whether the synapse loss is related to cognitive decline in the ALS cases [44].

Synapse loss is the strongest correlate of cognitive decline in Alzheimer’s disease [54] and a recent meta-analysis highlighted presynaptic terminals as the major site of early pathogenesis in the disease [14]. Therefore, the loss of synapses from the frontal cortex of ALS patients described here fits well with the features of other disorders where cognitive decline is observed.

ALS mouse models, including the hSOD1^G93A^ and FUS^R521C^ models, have revealed pre-symptomatic spine loss in the motor cortex [18] and sensorimotor cortex [43], respectively. Intriguingly, in the hSOD1^G93A^ model, early pre-symptomatic loss of spines on pyramidal cells in the medial prefrontal cortex preceded changes in dendritic arborization [17]. The earliest onset of anatomical change occurred in the motor cortex, with such severe neuronal breakdown that cortical thinning was detected at pre-symptomatic time-points [17]. Thinning of the medial prefrontal cortex was only detected in adult diseased mice, by which time spine loss and dendritic changes were significant [17]. This temporal pattern of cortical breakdown observed in mouse models fits with our observations in human post-mortem tissue. We observed synapse loss in the prefrontal cortex without neuron loss, yet found significant cortical thinning in the ALS motor cortex. Furthermore, our CSF results suggest that similar levels of neuronal breakdown occurred across the brain of ALS and ALSci groups (Supp. Fig. 5). This is most likely due to the cortical thinning observed in the motor cortex and supports our finding that synapse loss in the frontal cortex of ALSci patients is not due to neuronal loss.

Two human post-mortem studies have found greater expression of TDP-43 aggregates in the frontal cortex of patients with cognitive decline, compared to cognitively unimpaired ALS patients [9, 13]. TDP-43 aggregates are often found in the brain of ALS patients, and a sequential staging of pathology progression through the central nervous system has been described [8]. It is believed that TDP-43 pathology may progress through synaptically connected brain regions [6, 16], in a similar manner to other neurodegenerative diseases [7], thus placing synapses at a crucial point in ALS pathogenesis. Furthermore, in a human TDP-43 (hTDP43) overexpressing mouse model, synaptophysin loss was noted in whole brain homogenates [33] suggesting that altered TDP-43 function may drive synapse loss. Intriguingly, four studies have described what appears to be “synaptic” labeling of pTDP-43 in the neuropil of human post-mortem cortex from ALS and FTD patients [4, 37, 44, 53]. A recent study described pTDP-43 puncta in very close proximity to synaptophysin puncta in human ALS brain [53], with the suggestion that small pTDP-43 aggregates may be accumulating in dendritic spines. However, another recent study suggests pTDP-43 puncta co-localize with synaptophysin [44], thus suggesting these small aggregates are in fact presynaptic. In support of this, we also noted punctate labeling of pTDP-43 in the neuropil of some of our ALS cases and found pTDP-43 puncta in a number of synapses in a subset of ALS cases (Fig. 1i, j). Given that synaptic accumulation of pathological proteins in Alzheimer’s disease and FTLD-tau animal models drives synapse loss [27–29, 51, 52], it is possible that a similar build-up of pTDP-43 in the ALS synapse may initiate synapse breakdown. An alternative explanation is the phagocytosis of synaptic components by pathologically active glial cells, as recently described in Alzheimer’s disease [23]. In support of this, we recently observed that when TDP-43 is specifically knocked out in microglial cells, they adopt a hyper-phagocytic phenotype. The microglial cells actively phagocytozed synapses, resulting in a significantly lower synaptic density in the cortex of these transgenic mice compared to controls [39]. Furthermore, in a human cohort of ALS patients, pTDP-43 positive cases had significantly higher expression of the phagocytic microglial marker CD-68 [39]. Interestingly, in our current study, we found the same result in the frontal cortex of ALS patients. Those with pTDP-43 in the frontal cortex had a higher CD-68 burden than ALS cases without pTDP-43 (pTDP-43-positive *n* = 14, pTDP-43-negative *n* = 20; 2-tailed unpaired *t* test; *p* = 0.028). However, CD68 expression did not correlate with synaptic density (Pearson *r* = − 0.012; *p* = 0.95) and thus further work is required to understand the role of phagocytic microglia in synapse loss.

We found no direct association between the presence of the dementia-associated pathologies, beta-amyloid and pTau in the frontal cortex and lower ECAS score (Fig. 2). This is in agreement with recent studies showing no association between Alzheimer’s pathology and cognition in ALS [9, 42]. However, it is interesting to note that 5 out of the 7 ALSci cases were positive for pTau and 5 of the 7 pTau + ve cases were ALSci. This finding aligns with the proposed model that certain forms of pTau in the frontal cortex may associate with cognitive decline in ALS [50, 59, 60] and warrants further study once more cases become available to us. One of the limitations of this study is the small number of cognitively impaired patients who have donated brain tissue for these synaptic analyses. As more people donate tissue, we may be able to further dissect the contributions of pathological protein accumulation within synapses and genetic contributors to synaptic loss and cognitive decline. Another potential confound in the study is that the control group prepared for array tomography imaging was significantly older than the ALS group (Table 1). However, since subtle age-related changes in synapse loss have been reported [36], we predict that this age discrepancy would make the detection of synapse loss in ALS more difficult and thus are confident that this age difference did not lessen the main finding of synapse loss in frontal cortex of ALS cases, which is worse with cognitive impairment. In support of this idea, we did not detect any correlation between age and synapse density in our data (Pearson *r* = 0.23, *p* = 0.23).

In summary, we have combined two high-resolution imaging techniques to reveal synapse loss in the frontal cortex of ALS patients. We show that ALS patients with cognitive impairment have lower synaptic densities than controls. Synapse density did not differ in the primary motor cortex between ALS and controls, most likely due to the significant cortical thinning we observed in the ALS motor cortex. These findings significantly advance our understanding of ALS-associated neuropathology and identify the underlying neurobiological substrate for cognitive decline in ALS. Further, these results suggest potential synaptic biomarkers for predicting which ALS patients will experience cognitive changes, and point to the potential of synapse-directed therapeutics to combat both the cognitive and potentially also the motor symptoms of ALS.

## Electronic supplementary material

Below is the link to the electronic supplementary material.

Supplementary material 1 (XLSX 30 kb)

Supplementary material 2 (PPTX 450 kb)

## References

[1] Abrahams S, Goldstein LH, Simmons A, Brammer M, Williams SC, Giampietro V, Leigh PN, Williams SCR (2004). Word retrieval in amyotrophic lateral sclerosis: a functional magnetic resonance imaging study. Brain.

[2] Abrahams S, Goldstein LH, Suckling J, Ng V, Simmons A, Chitnis X, Atkins L, Williams SC, Leigh PN, Abrahams S (2005). Frontotemporal white matter changes in amyotrophic lateral sclerosis. J Neurol.

[3] Abrahams S, Newton J, Niven E, Foley J, Bak TH (2014). Screening for cognition and behaviour changes in ALS. Amyotroph Later Scler Frontotemporal Degener.

[4] Bigio EH, Weintraub S, Rademakers R, Baker M, Ahmadian SS, Rademaker A, Weitner BB, Mao Q, Lee KH, Mishra M (2013). Frontotemporal lobar degeneration with TDP-43 proteinopathy and chromosome 9p repeat expansion in C9ORF72: clinicopathologic correlation. Neuropathology.

[5] Black HA, Leighton DJ, Cleary EM, Rose E, Stephenson L, Colville S, Ross D, Warner J, Porteous M, Gorrie GH (2017). Genetic epidemiology of motor neuron disease-associated variants in the Scottish population. Neurobiol Aging.

[6] Braak H, Ludolph AC, Neumann M, Ravits J, Del Tredici K (2017). Pathological TDP-43 changes in Betz cells differ from those in bulbar and spinal alpha-motoneurons in sporadic amyotrophic lateral sclerosis. Acta Neuropathol.

[7] Brettschneider J, Del Tredici K, Lee VM, Trojanowski JQ (2015). Spreading of pathology in neurodegenerative diseases: a focus on human studies. Nat Rev Neurosci.

[8] Brettschneider J, Del Tredici K, Toledo JB, Robinson JL, Irwin DJ, Grossman M, Suh E, Van Deerlin VM, Wood EM, Baek Y (2013). Stages of pTDP-43 pathology in amyotrophic lateral sclerosis. Ann Neurol.

[9] Brettschneider J, Libon DJ, Toledo JB, Xie SX, McCluskey L, Elman L, Geser F, Lee VM, Grossman M, Trojanowski JQ (2012). Microglial activation and TDP-43 pathology correlate with executive dysfunction in amyotrophic lateral sclerosis. Acta Neuropathol.

[10] Brooks BR, Miller RG, Swash M, Munsat TL (1998) El Escorial revisited: revised criteria for the diagnosis of amyotrophic lateral sclerosis. World Federation of Neurology Research Group on Motor Neuron Diseases A consensus conference, City 10.1080/146608200300079536 11464847

[11] Canosa A, Pagani M, Cistaro A, Montuschi A, Iazzolino B, Fania P, Cammarosano S, Ilardi A, Moglia C, Calvo A (2016). 18F-FDG-PET correlates of cognitive impairment in ALS. Neurology.

[12] Cleary EM, Pal S, Azam T, Moore DJ, Swingler R, Gorrie G, Stephenson L, Colville S, Chandran S, Porteous M (2016). Improved PCR based methods for detecting C9orf72 hexanucleotide repeat expansions. Mol Cell Probes.

[13] Cykowski MD, Powell SZ, Peterson LE, Appel JW, Rivera AL, Takei H, Chang E, Appel SH (2017). Clinical significance of TDP-43 neuropathology in amyotrophic lateral sclerosis. J Neuropathol Exp Neurol.

[14] de Wilde MC, Overk CR, Sijben JW, Masliah E (2016). Meta-analysis of synaptic pathology in Alzheimer’s disease reveals selective molecular vesicular machinery vulnerability. Alzheimers Dement.

[15] Elamin M, Phukan J, Bede P, Jordan N, Byrne S, Pender N, Hardiman O (2011). Executive dysfunction is a negative prognostic indicator in patients with ALS without dementia. Neurology.

[16] Feiler MS, Strobel B, Freischmidt A, Helferich AM, Kappel J, Brewer BM, Li D, Thal DR, Walther P, Ludolph AC (2015). TDP-43 is intercellularly transmitted across axon terminals. J Cell Biol.

[17] Fogarty MJ, Mu EW, Noakes PG, Lavidis NA, Bellingham MC (2016). Marked changes in dendritic structure and spine density precede significant neuronal death in vulnerable cortical pyramidal neuron populations in the SOD1 (G93A) mouse model of amyotrophic lateral sclerosis. Acta Neuropathol Commun.

[18] Fogarty MJ, Noakes PG, Bellingham MC (2015). Motor cortex layer V pyramidal neurons exhibit dendritic regression, spine loss, and increased synaptic excitation in the presymptomatic hSOD1(G93A) mouse model of amyotrophic lateral sclerosis. J Neurosci.

[19] Gillingwater TH, Ingham CA, Parry KE, Wright AK, Haley JE, Wishart TM, Arbuthnott GW, Ribchester RR (2006). Delayed synaptic degeneration in the CNS of Wlds mice after cortical lesion. Brain.

[20] Goldstein LH, Abrahams S (2013). Changes in cognition and behaviour in amyotrophic lateral sclerosis: nature of impairment and implications for assessment. Lancet Neurol.

[21] Henstridge CM, Jackson RJ, Kim JM, Herrmann AG, Wright AK, Harris SE, Bastin ME, Starr JM, Wardlaw J, Gillingwater TH (2015). Post-mortem brain analyses of the Lothian Birth Cohort 1936: extending lifetime cognitive and brain phenotyping to the level of the synapse. Acta Neuropathol Commun.

[22] Henstridge CM, Pickett E, Spires-Jones TL (2016). Synaptic pathology: a shared mechanism in neurological disease. Ageing Res Rev.

[23] Hong S, Beja-Glasser VF, Nfonoyim BM, Frouin A, Li S, Ramakrishnan S, Merry KM, Shi Q, Rosenthal A, Barres BA (2016). Complement and microglia mediate early synapse loss in Alzheimer mouse models. Science.

[24] Hudson AJ (1981). Amyotrophic lateral sclerosis and its association with dementia, parkinsonism and other neurological disorders: a review. Brain.

[25] Ince PG, Slade J, Chinnery RM, McKenzie J, Royston C, Roberts GW, Shaw PJ (1995). Quantitative study of synaptophysin immunoreactivity of cerebral cortex and spinal cord in motor neuron disease. J Neuropathol Exp Neurol.

[26] Kay KR, Smith C, Wright AK, Serrano-Pozo A, Pooler AM, Koffie R, Bastin ME, Bak TH, Abrahams S, Kopeikina KJ (2013). Studying synapses in human brain with array tomography and electron microscopy. Nat Protoc.

[27] Kopeikina KJ, Carlson GA, Pitstick R, Ludvigson AE, Peters A, Luebke JI, Koffie RM, Frosch MP, Hyman BT, Spires-Jones TL (2011). Tau accumulation causes mitochondrial distribution deficits in neurons in a mouse model of tauopathy and in human Alzheimer’s disease brain. Am J Pathol.

[28] Kopeikina KJ, Polydoro M, Tai HC, Yaeger E, Carlson GA, Pitstick R, Hyman BT, Spires-Jones TL (2013). Synaptic alterations in the rTg4510 mouse model of tauopathy. J Comp Neurol.

[29] Kopeikina KJ, Wegmann S, Pitstick R, Carlson GA, Bacskai BJ, Betensky RA, Hyman BT, Spires-Jones TL (2013). Tau causes synapse loss without disrupting calcium homeostasis in the rTg4510 model of tauopathy. PLoS One.

[30] Ling SC, Polymenidou M, Cleveland DW (2013). Converging mechanisms in ALS and FTD: disrupted RNA and protein homeostasis. Neuron.

[31] Lulé D, Burkhardt C, Abdulla S, Böhm S, Kollewe K, Uttner I, Abrahams S, Bak TH, Petri S, Weber M (2015). The Edinburgh Cognitive and Behavioural Amyotrophic Lateral Sclerosis Screen: a cross-sectional comparison of established screening tools in a German–Swiss population. Amyotroph Later Scler Frontotemporal Degener.

[32] Mackenzie IR, Bigio EH, Ince PG, Geser F, Neumann M, Cairns NJ, Kwong LK, Forman MS, Ravits J, Stewart H (2007). Pathological TDP-43 distinguishes sporadic amyotrophic lateral sclerosis from amyotrophic lateral sclerosis with SOD1 mutations. Ann Neurol.

[33] Medina DX, Orr ME, Oddo S (2014). Accumulation of C-terminal fragments of transactive response DNA-binding protein 43 leads to synaptic loss and cognitive deficits in human TDP-43 transgenic mice. Neurobiol Aging.

[34] Menke RAL, Agosta F, Grosskreutz J, Filippi M, Turner MR (2017). Neuroimaging endpoints in amyotrophic lateral sclerosis. Neurotherapeutics.

[35] Micheva KD, Busse B, Weiler NC, O’Rourke N, Smith SJ (2010). Single-synapse analysis of a diverse synapse population: proteomic imaging methods and markers. Neuron.

[36] Morrison JH, Baxter MG (2012). The ageing cortical synapse: hallmarks and implications for cognitive decline. Nat Rev Neurosci.

[37] Murray ME, DeJesus-Hernandez M, Rutherford NJ, Baker M, Duara R, Graff-Radford NR, Wszolek ZK, Ferman T, Josephs KA, Boylan KB (2011). Clinical and neuropathologic heterogeneity of c9FTD/ALS associated with hexanucleotide repeat expansion in C9ORF72. Acta Neuropathol.

[38] Niven E, Newton J, Foley J, Colville S, Swingler R, Chandran S, Bak TH, Abrahams S (2015). Validation of the Edinburgh Cognitive and Behavioural Amyotrophic Lateral Sclerosis Screen (ECAS): a cognitive tool for motor disorders. Amyotroph Later Scler Frontotemporal Degener.

[39] Paolicelli RC, Jawaid A, Henstridge CM, Valeri A, Merlini M, Robinson JL, Lee EB, Rose J, Appel S, Lee VM (2017). TDP-43 depletion in microglia promotes amyloid clearance but also induces synapse loss. Neuron.

[40] Philips T, Rothstein J (2014). Glial cells in amyotrophic lateral sclerosis. Exp Neurol.

[41] Poletti BSF, Carelli L, Madotto F, Lafronza A, Faini A, Monti A, Zago S, Calini D, Tiloca C, Doretti A, Verde F, Ratti A, Ticozzi N, Abrahams S, Silani V (2016). The validation of the Italian Edinburgh Cognitive and Behavioural ALS Screen (ECAS). Amyotroph Later Scler Frontotemporal Degener.

[42] Prudlo J, Konig J, Schuster C, Kasper E, Buttner A, Teipel S, Neumann M (2016). TDP-43 pathology and cognition in ALS: a prospective clinicopathologic correlation study. Neurology.

[43] Qiu H, Lee S, Shang Y, Wang WY, Au KF, Kamiya S, Barmada SJ, Finkbeiner S, Lui H, Carlton CE (2014). ALS-associated mutation FUS-R521C causes DNA damage and RNA splicing defects. J Clin Invest.

[44] Riku Y, Watanabe H, Yoshida M, Mimuro M, Iwasaki Y, Masuda M, Ishigaki S, Katsuno M, Sobue G (2017). Pathologic involvement of glutamatergic striatal inputs from the cortices in TAR DNA-binding protein 43 kDa-related frontotemporal lobar degeneration and amyotrophic lateral sclerosis. J Neuropathol Exp Neurol.

[45] Rothstein JD, Van Kammen M, Levey AI, Martin LJ, Kuncl RW (1995). Selective loss of glial glutamate transporter GLT-1 in amyotrophic lateral sclerosis. Ann Neurol.

[46] Samarasekera N, Salman RA, Huitinga I, Klioueva N, McLean CA, Kretzschmar H, Smith C, Ironside JW (2013). Brain banking for neurological disorders. Lancet Neurol.

[47] Schuster C, Kasper E, Dyrba M, Machts J, Bittner D, Kaufmann J, Mitchell AJ, Benecke R, Teipel S, Vielhaber S (2014). Cortical thinning and its relation to cognition in amyotrophic lateral sclerosis. Neurobiol Aging.

[48] Spires-Jones TL, Hyman BT (2014). The intersection of amyloid beta and tau at synapses in Alzheimer’s disease. Neuron.

[49] Strong MJ, Abrahams S, Goldstein LH, Woolley S, McLaughlin P, Snowden J, Mioshi E, Roberts-South A, Benatar M, HortobaGyi T (2017). Amyotrophic lateral sclerosis-frontotemporal spectrum disorder (ALS-FTSD): revised diagnostic criteria. Amyotroph Later Scler Frontotemporal Degener.

[50] Strong MJ, Yang W, Strong WL, Leystra-Lantz C, Jaffe H, Pant HC (2006). Tau protein hyperphosphorylation in sporadic ALS with cognitive impairment. Neurology.

[51] Tai HC, Serrano-Pozo A, Hashimoto T, Frosch MP, Spires-Jones TL, Hyman BT (2012). The synaptic accumulation of hyperphosphorylated tau oligomers in Alzheimer disease is associated with dysfunction of the ubiquitin-proteasome system. Am J Pathol.

[52] Tai HC, Wang BY, Serrano-Pozo A, Frosch MP, Spires-Jones TL, Hyman BT (2014). Frequent and symmetric deposition of misfolded tau oligomers within presynaptic and postsynaptic terminals in Alzheimer’s disease. Acta Neuropathol Commun.

[53] Takeuchi R, Tada M, Shiga A, Toyoshima Y, Konno T, Sato T, Nozaki H, Kato T, Horie M, Shimizu H (2016). Heterogeneity of cerebral TDP-43 pathology in sporadic amyotrophic lateral sclerosis: evidence for clinico-pathologic subtypes. Acta Neuropathol Commun.

[54] Terry RD, Masliah E, Salmon DP, Butters N, DeTeresa R, Hill R, Hansen LA, Katzman R (1991). Physical basis of cognitive alterations in Alzheimer’s disease: synapse loss is the major correlate of cognitive impairment. Ann Neurol.

[55] Thevenaz P, Ruttimann UE, Unser M (1998). A pyramid approach to subpixel registration based on intensity. IEEE Trans Image Process.

[56] Turner MR, Agosta F, Bede P, Govind V, Lule D, Verstraete E (2012). Neuroimaging in amyotrophic lateral sclerosis. Biomark Med.

[57] Turner MR, Hardiman O, Benatar M, Brooks BR, Chio A, de Carvalho M, Ince PG, Lin C, Miller RG, Mitsumoto H (2013). Controversies and priorities in amyotrophic lateral sclerosis. Lancet Neurol.

[58] Xu Z, Alruwaili ARS, Henderson RD, McCombe PA (2017). Screening for cognitive and behavioural impairment in amyotrophic lateral sclerosis: frequency of abnormality and effect on survival. J Neurol Sci.

[59] Yang W, Ang LC, Strong MJ (2005). Tau protein aggregation in the frontal and entorhinal cortices as a function of aging. Brain Res Dev Brain Res.

[60] Yang W, Sopper MM, Leystra-Lantz C, Strong MJ (2003). Microtubule-associated tau protein positive neuronal and glial inclusions in ALS. Neurology.

