# Supplementary material for: Synapse loss in the prefrontal cortex is associated with cognitive decline in amyotrophic lateral sclerosis
Source: Acta Neuropathol. 2017 Dec 22;135(2):213–26. doi: 10.1007/s00401-017-1797-4 (PMC5773656; doi:10.1007/s00401-017-1797-4)
Supplement: Supplementary file 2 — Supplementary material 2 (PPTX 450 kb) [file 401_2017_1797_MOESM2_ESM.pptx]

## Slide 1
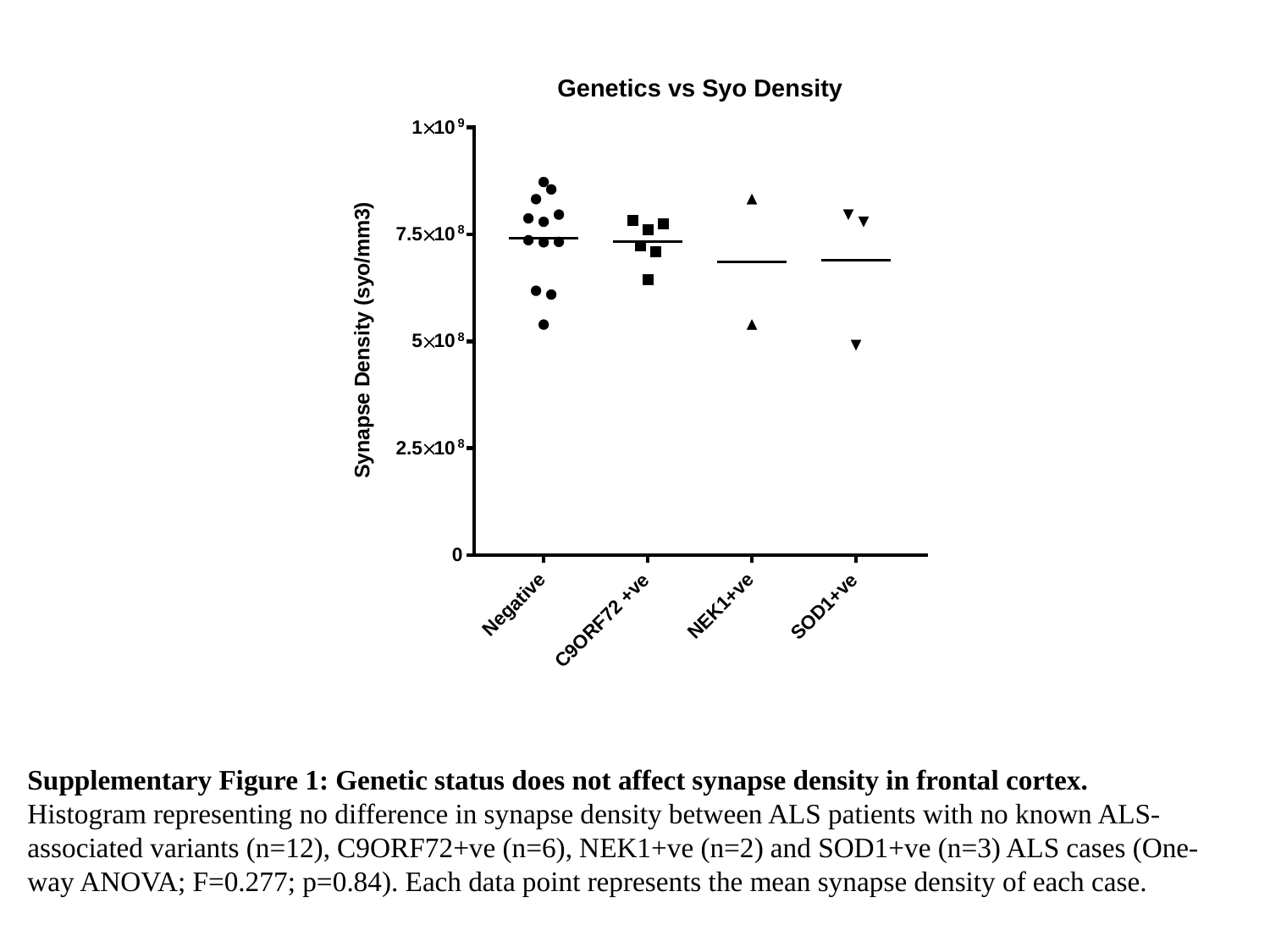

Supplementary Figure 1: Genetic status does not affect synapse density in frontal cortex.
Histogram representing no difference in synapse density between ALS patients with no known ALS-associated variants (n=12), C9ORF72+ve (n=6), NEK1+ve (n=2) and SOD1+ve (n=3) ALS cases (One-way ANOVA; F=0.277; p=0.84). Each data point represents the mean synapse density of each case.

## Slide 2
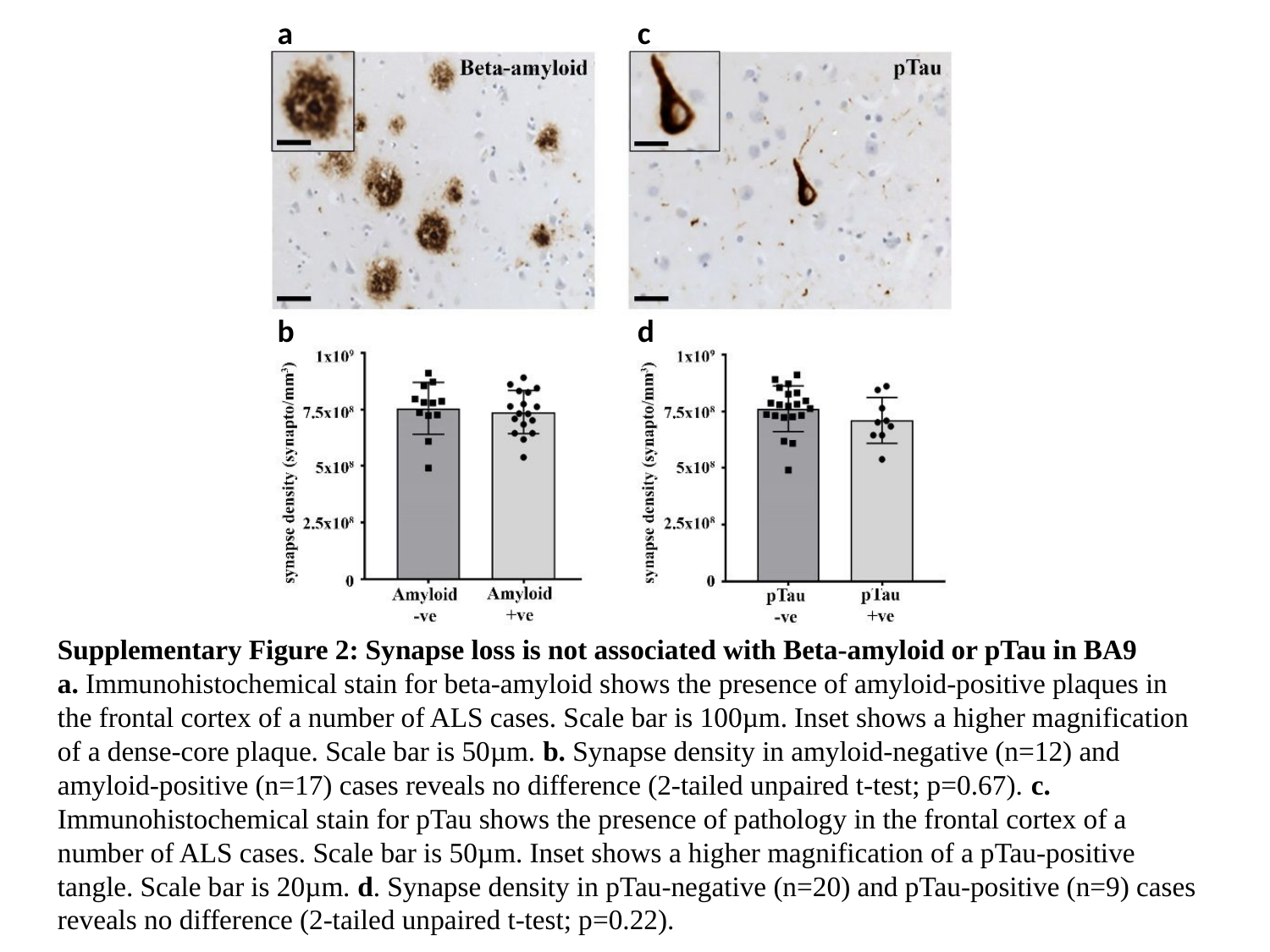

a
c
b
d
Supplementary Figure 2: Synapse loss is not associated with Beta-amyloid or pTau in BA9
a. Immunohistochemical stain for beta-amyloid shows the presence of amyloid-positive plaques in the frontal cortex of a number of ALS cases. Scale bar is 100µm. Inset shows a higher magnification of a dense-core plaque. Scale bar is 50µm. b. Synapse density in amyloid-negative (n=12) and amyloid-positive (n=17) cases reveals no difference (2-tailed unpaired t-test; p=0.67). c. Immunohistochemical stain for pTau shows the presence of pathology in the frontal cortex of a number of ALS cases. Scale bar is 50µm. Inset shows a higher magnification of a pTau-positive tangle. Scale bar is 20µm. d. Synapse density in pTau-negative (n=20) and pTau-positive (n=9) cases reveals no difference (2-tailed unpaired t-test; p=0.22).

## Slide 3
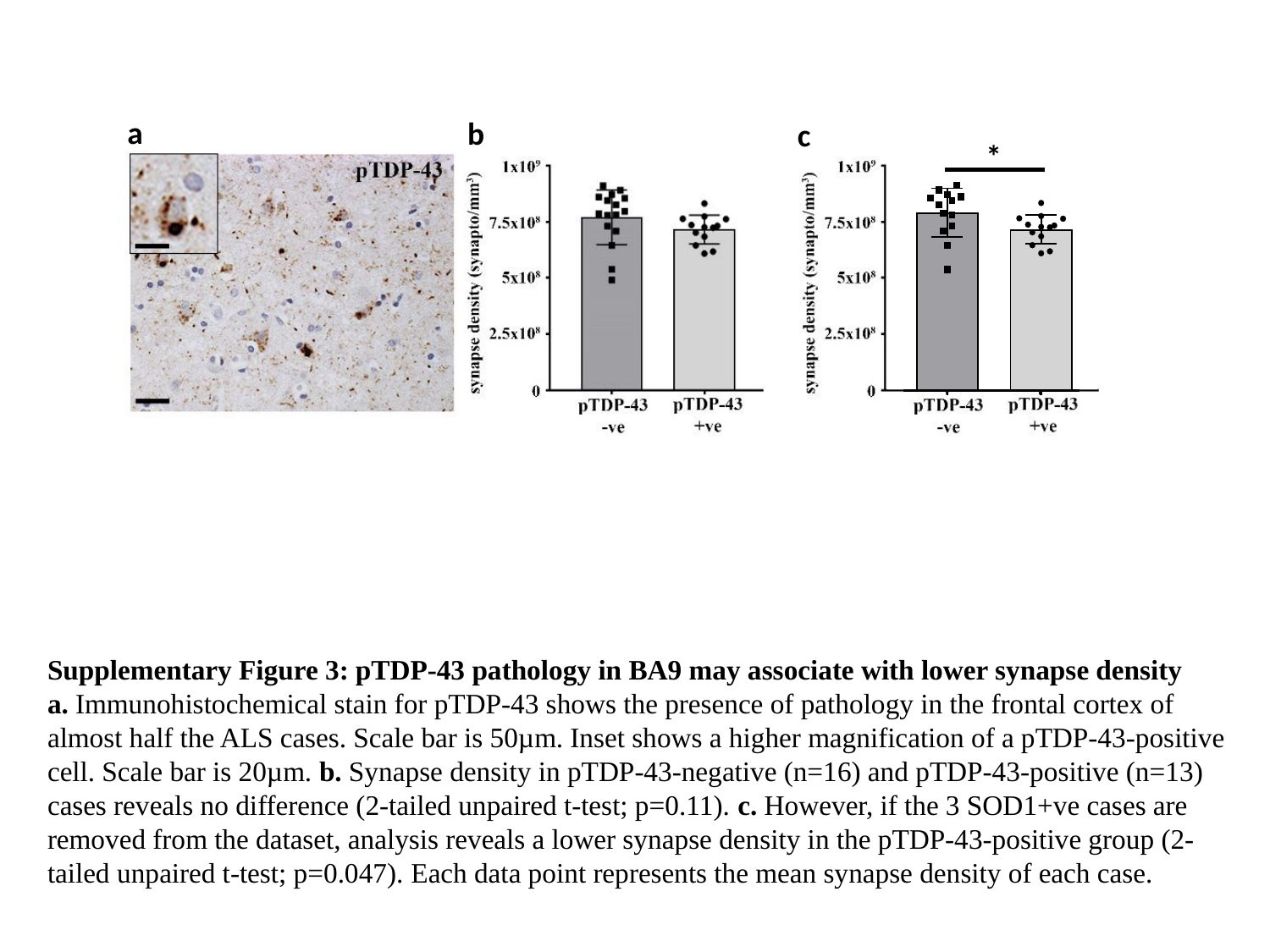

a
b
c
*
Supplementary Figure 3: pTDP-43 pathology in BA9 may associate with lower synapse density
a. Immunohistochemical stain for pTDP-43 shows the presence of pathology in the frontal cortex of almost half the ALS cases. Scale bar is 50µm. Inset shows a higher magnification of a pTDP-43-positive cell. Scale bar is 20µm. b. Synapse density in pTDP-43-negative (n=16) and pTDP-43-positive (n=13) cases reveals no difference (2-tailed unpaired t-test; p=0.11). c. However, if the 3 SOD1+ve cases are removed from the dataset, analysis reveals a lower synapse density in the pTDP-43-positive group (2-tailed unpaired t-test; p=0.047). Each data point represents the mean synapse density of each case.

## Slide 4
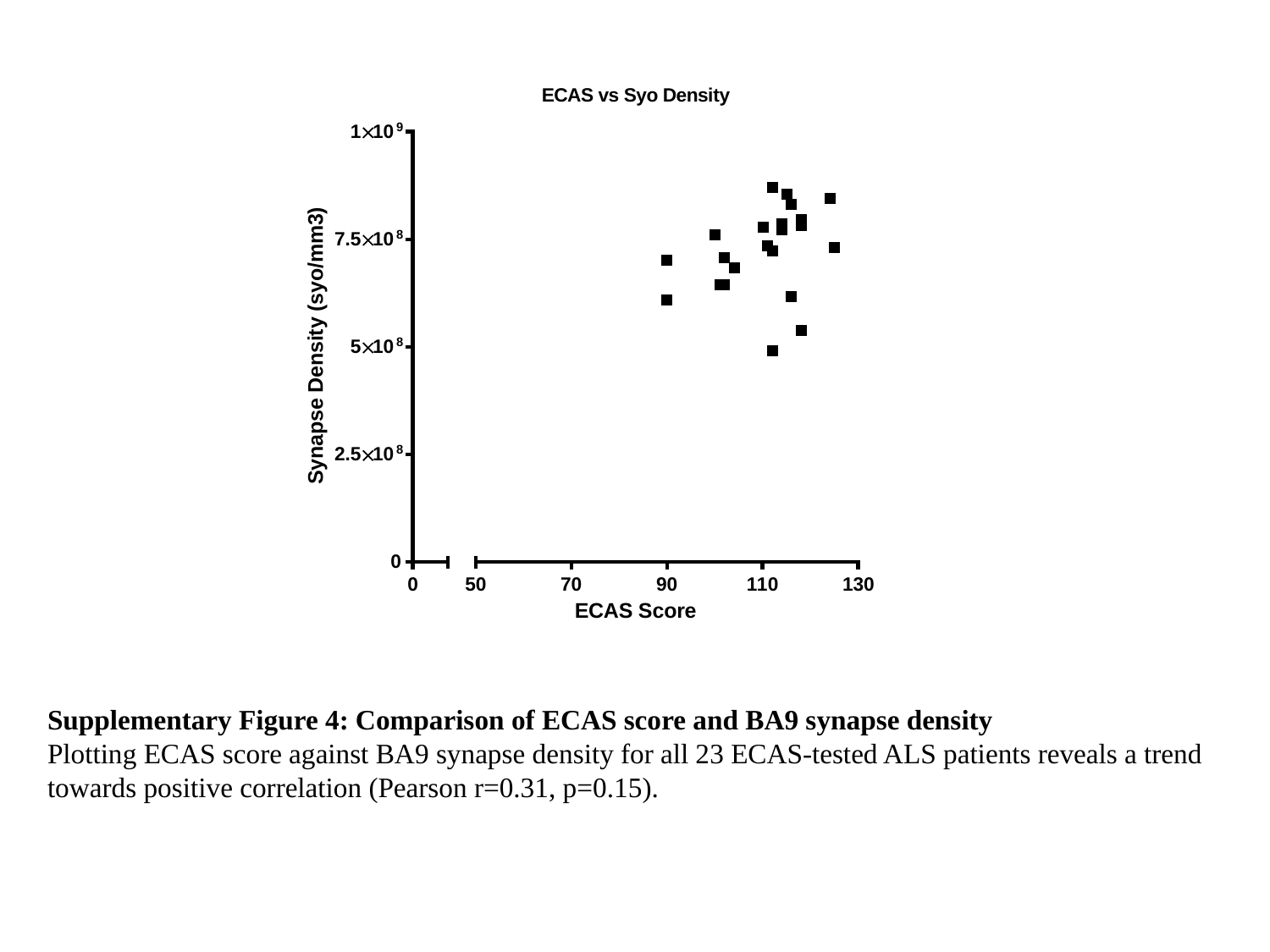

Supplementary Figure 4: Comparison of ECAS score and BA9 synapse density
Plotting ECAS score against BA9 synapse density for all 23 ECAS-tested ALS patients reveals a trend towards positive correlation (Pearson r=0.31, p=0.15).

## Slide 5
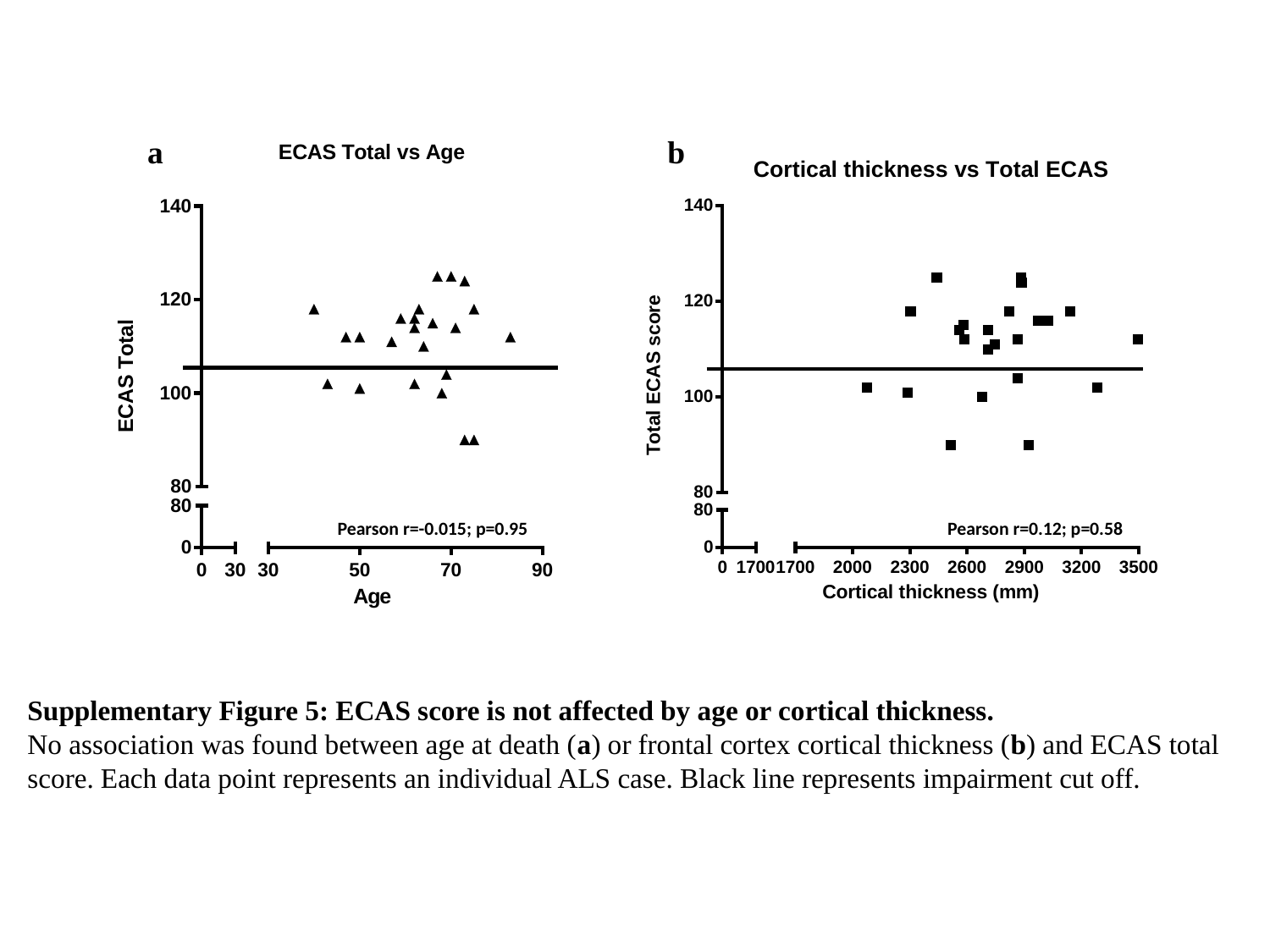

a
b
Pearson r=-0.015; p=0.95
Pearson r=0.12; p=0.58
Supplementary Figure 5: ECAS score is not affected by age or cortical thickness.
No association was found between age at death (a) or frontal cortex cortical thickness (b) and ECAS total score. Each data point represents an individual ALS case. Black line represents impairment cut off.

## Slide 6
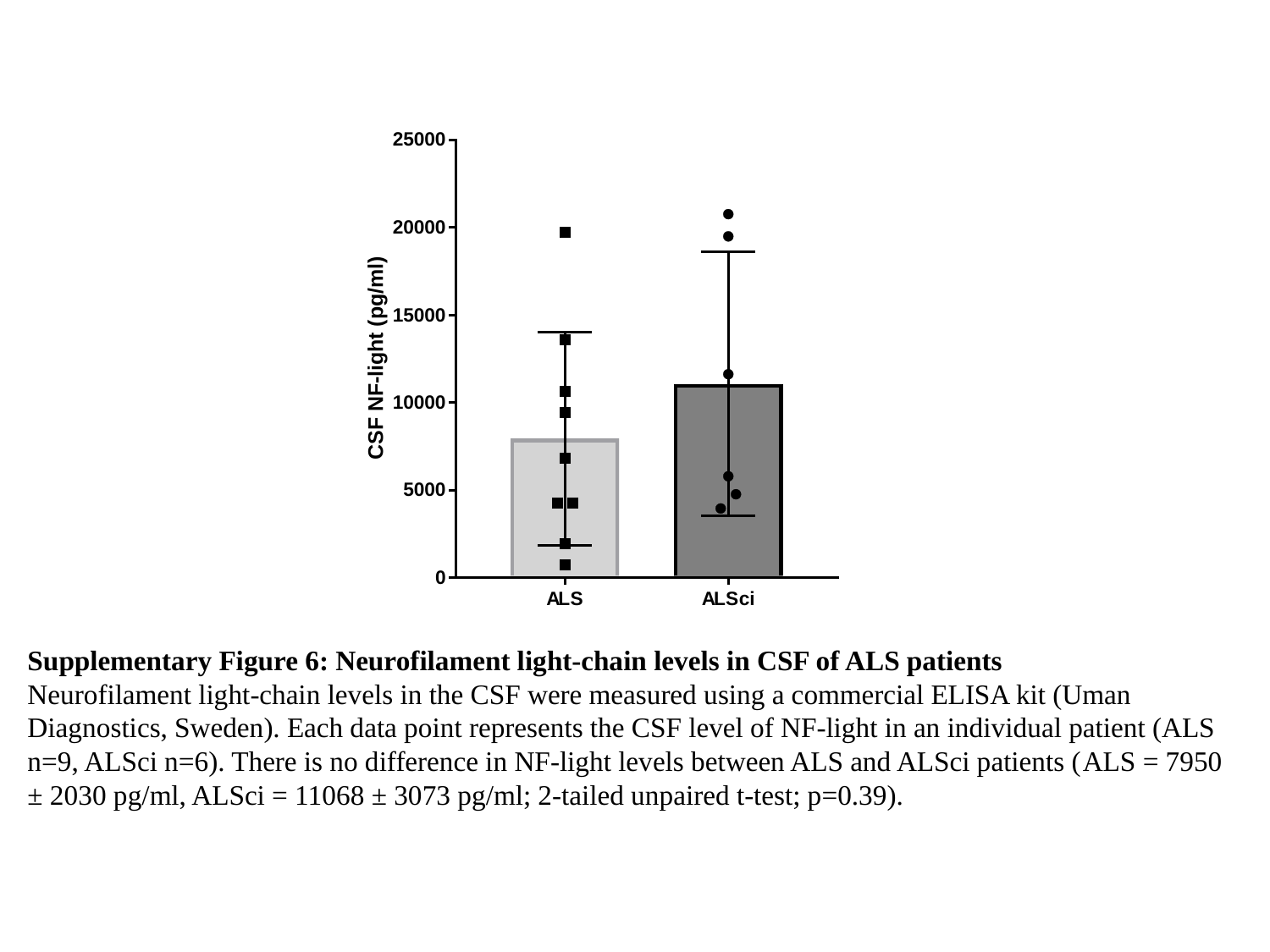

Supplementary Figure 6: Neurofilament light-chain levels in CSF of ALS patients
Neurofilament light-chain levels in the CSF were measured using a commercial ELISA kit (Uman Diagnostics, Sweden). Each data point represents the CSF level of NF-light in an individual patient (ALS n=9, ALSci n=6). There is no difference in NF-light levels between ALS and ALSci patients (ALS = 7950 ± 2030 pg/ml, ALSci = 11068 ± 3073 pg/ml; 2-tailed unpaired t-test; p=0.39).

## Slide 7
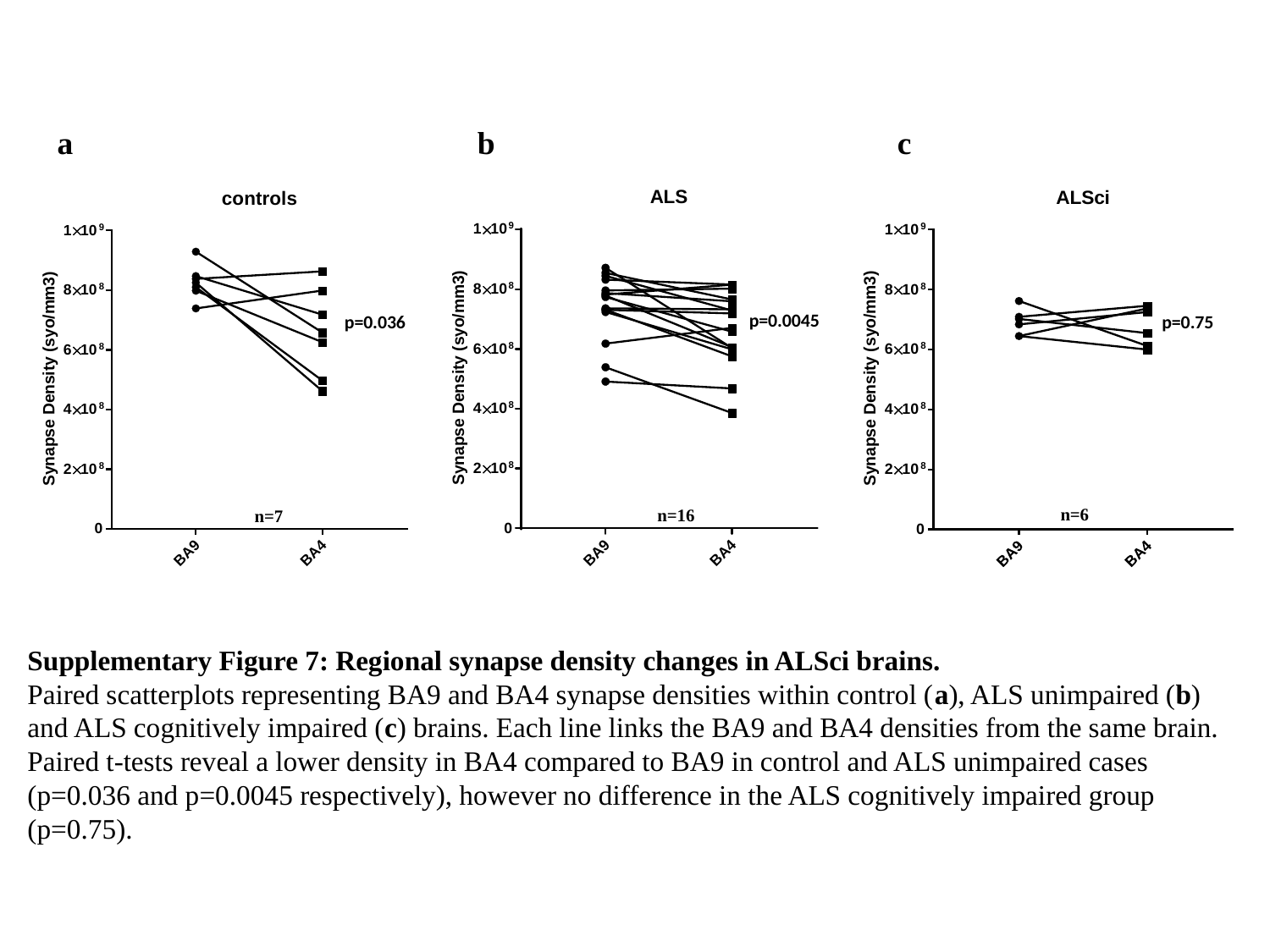

a
b
c
p=0.0045
p=0.036
p=0.75
n=6
n=16
n=7
Supplementary Figure 7: Regional synapse density changes in ALSci brains.
Paired scatterplots representing BA9 and BA4 synapse densities within control (a), ALS unimpaired (b) and ALS cognitively impaired (c) brains. Each line links the BA9 and BA4 densities from the same brain. Paired t-tests reveal a lower density in BA4 compared to BA9 in control and ALS unimpaired cases (p=0.036 and p=0.0045 respectively), however no difference in the ALS cognitively impaired group (p=0.75).

## Slide 8
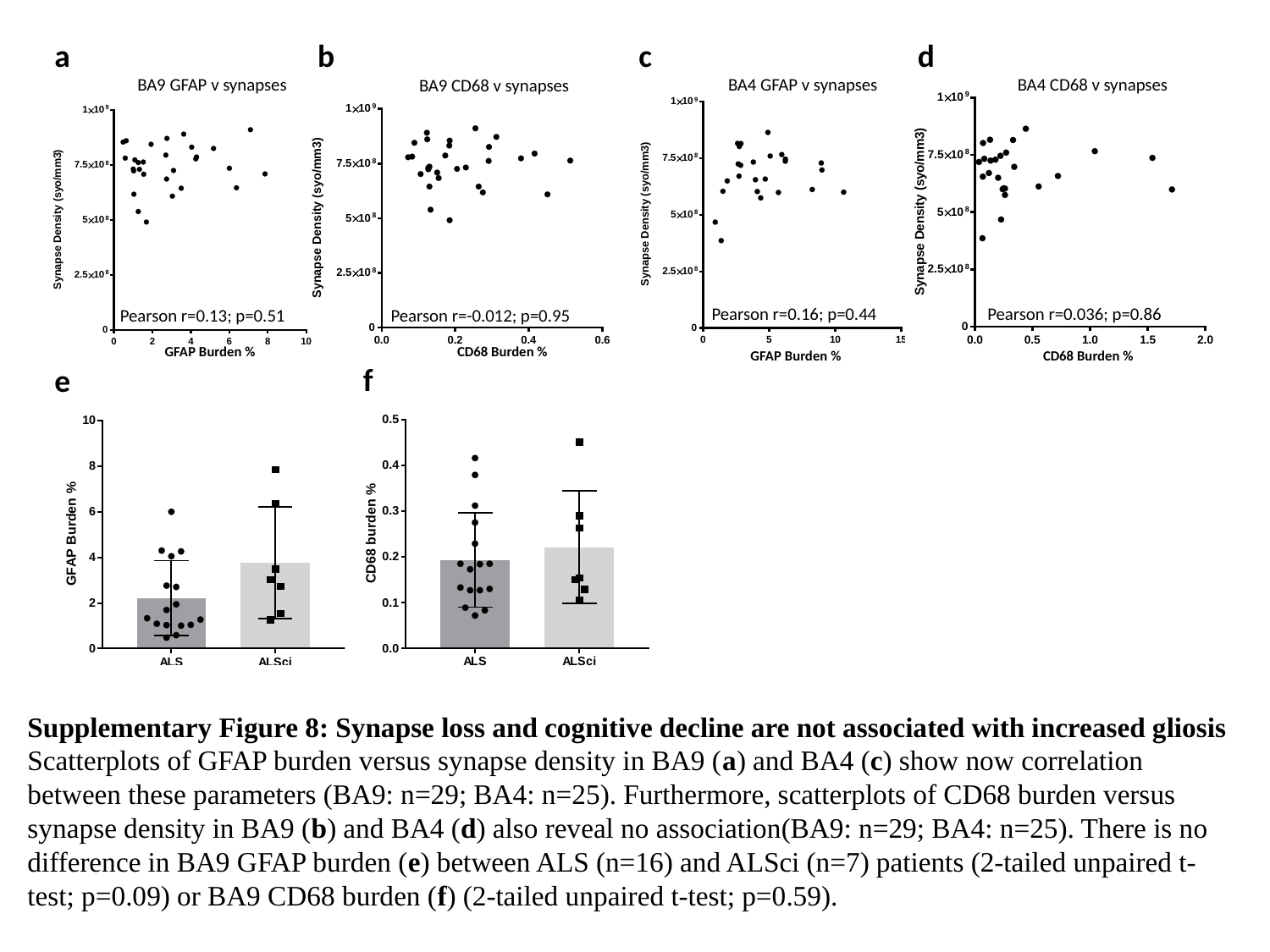

a
b
c
d
BA4 GFAP v synapses
BA9 GFAP v synapses
BA4 CD68 v synapses
BA9 CD68 v synapses
Pearson r=0.16; p=0.44
Pearson r=0.036; p=0.86
Pearson r=0.13; p=0.51
Pearson r=-0.012; p=0.95
GFAP Burden %
CD68 Burden %
GFAP Burden %
CD68 Burden %
f
e
Supplementary Figure 8: Synapse loss and cognitive decline are not associated with increased gliosis
Scatterplots of GFAP burden versus synapse density in BA9 (a) and BA4 (c) show now correlation between these parameters (BA9: n=29; BA4: n=25). Furthermore, scatterplots of CD68 burden versus synapse density in BA9 (b) and BA4 (d) also reveal no association(BA9: n=29; BA4: n=25). There is no difference in BA9 GFAP burden (e) between ALS (n=16) and ALSci (n=7) patients (2-tailed unpaired t-test; p=0.09) or BA9 CD68 burden (f) (2-tailed unpaired t-test; p=0.59).
